# Effects of brewers’ spent grain protein hydrolysates on gas production, ruminal fermentation characteristics, microbial protein synthesis and microbial community in an artificial rumen fed a high grain diet

**DOI:** 10.1186/s40104-020-00531-5

**Published:** 2021-01-04

**Authors:** Tao Ran, Long Jin, Ranithri Abeynayake, Atef Mohamed Saleem, Xiumin Zhang, Dongyan Niu, Lingyun Chen, Wenzhu Yang

**Affiliations:** 1 Agriculture and Agri-Food of Canada, Lethbridge Research and Development Centre, Lethbridge, AB T1J 4B1 Canada; 2 Key Laboratory for Agro-Ecological Processes in Subtropical Region, Institute of Subtropical Agriculture, Chinese Academy of Sciences, Changsha, 410125 Hunan China; 3 College of Veterinary Medicine, University of Calgary, 2500 University Dr. NW, Calgary, AB T2N 1N4 Canada; 4 Department of Agricultural, Food and Nutritional Science, University of Alberta, Edmonton, T6G 2P5 Canada; 5 Department of Animal and Poultry Production, Faculty of Agriculture, South Valley University, Qena, 83523 Egypt

**Keywords:** Antioxidant peptide, Brewers’ spent grain, Fermentation, Hydrogen production, Methane production, Protein hydrolysates, RUSITEC

## Abstract

**Background:**

Brewers’ spent grain (BSG) typically contains 20% – 29% crude protein (CP) with high concentrations of glutamine, proline and hydrophobic and non-polar amino acid, making it an ideal material for producing value-added products like bioactive peptides which have antioxidant properties. For this study, protein was extracted from BSG, hydrolyzed with 1% alcalase and flavourzyme, with the generated protein hydrolysates (AlcH and FlaH) showing antioxidant activities. This study evaluated the effects of AlcH and FlaH on gas production, ruminal fermentation characteristics, nutrient disappearance, microbial protein synthesis and microbial community using an artificial rumen system (RUSITEC) fed a high-grain diet.

**Results:**

As compared to the control of grain only, supplementation of FlaH decreased (*P* < 0.01) disappearances of dry matter (DM), organic matter (OM), CP and starch, without affecting fibre disappearances; while AlcH had no effect on nutrient disappearance. Neither AlcH nor FlaH affected gas production or VFA profiles, however they increased (*P* < 0.01) NH_3_-N and decreased (*P* < 0.01) H_2_ production. Supplementation of FlaH decreased (*P* < 0.01) the percentage of CH_4_ in total gas and dissolved-CH_4_ (dCH_4_) in dissolved gas. Addition of monensin reduced (*P* < 0.01) disappearance of nutrients, improved fermentation efficiency and reduced CH_4_ and H_2_ emissions. Total microbial nitrogen production was decreased (*P* < 0.05) but the proportion of feed particle associated (FPA) bacteria was increased with FlaH and monensin supplementation. Numbers of OTUs and Shannon diversity indices of FPA microbial community were unaffected by AlcH and FlaH; whereas both indices were reduced (*P* < 0.05) by monensin. Taxonomic analysis revealed no effect of AlcH and FlaH on the relative abundance (RA) of bacteria at phylum level, whereas monensin reduced (*P* < 0.05) the RA of Firmicutes and Bacteroidetes and enhanced Proteobacteria. Supplementation of FlaH enhanced (*P* < 0.05) the RA of genus *Prevotella*, reduced *Selenomonas*, *Shuttleworthia*, *Bifidobacterium* and *Dialister* as compared to control; monensin reduced (*P* < 0.05) RA of genus *Prevotella* but enhaced *Succinivibrio*.

**Conclusions:**

The supplementation of FlaH in high-grain diets may potentially protect CP and starch from ruminal degradation, without adversely affecting fibre degradation and VFA profiles. It also showed promising effects on reducing CH_4_ production by suppressing H_2_ production. Protein enzymatic hydrolysates from BSG using flavourzyme showed potential application to high value-added bio-products.

**Supplementary Information:**

The online version contains supplementary material available at 10.1186/s40104-020-00531-5.

## Background

Brewers’ spent grain (BSG) is the most abundant by-product in brewing industry, accounting for 85% of the total by-products generated [1]. Currently, the main application of BSG is as animal feed, especially for ruminants, as it contains up to 29% crude protein (CP) and up to 60% fiber on dry matter (DM) basis [2]. Low level inclusion of wet BSG in conventional finishing diets can maintain growth performance and meat quality of finishing beef cattle compared with conventional finishing diets [3]. However, there are some potential disadvantages when including BSG in high amounts in ruminant diets, such as reducing feed intake and increasing the incidence of liver abscesses [4], increasing the release of enteric pathogenic bacteria *E. coli* O157:H7 [5, 6] and increased excretion of N, P and S to the environment [7]. Furthermore, the utilization of BSG in animal production has been limited due to its high moisture and fermentable sugar contents, which make it unstable and easily spoiled due to enhanced microbial activity [1, 2]. As a result, its use to date has generally been limited to animal operations near breweries due to high energy costs of transportation [1]. Although drying is considered the most effective method for lowering storage and transportation costs, drying itself is an energetically expensive process [1, 8]. Therefore, due to the high yield but limited usage of BSG, there is an urgent need for developing new strategies to increase the feasibility of utilizing BSG in cattle production systems.

Nowadays, there is increasing interest in the study of by-products from the food processing industry as feed ingredients for animal production and also for extraction of value-added bioactive compounds, which can possibly be used as natural antimicrobials or antioxidants [9]. Barley is the primary grain source used in the brewing industry with the derived BSG being rich in phenolic compounds and hordein and glutelin proteins. Emerging evidence indicates that phenolic compounds in diet exhibit anti-inflammatory and antioxidant properties [8, 10]. The unique structural features of barley proteins coupled with high concentrations of glutamine and hydrophobic and non-polar residues, offer the possibility of producing bioactive peptides with strong antioxidant and antimicrobial properties upon proteolytic degradation using commercially available proteinase or alkali solutions [11, 12]. Bioactive peptides have a broad range of functions: antibacterial, anti-inflammatory, antioxidant and immune function enhancing. Both glutelin and hordein protein hydrolysates have been proven to having antioxidant activities [11, 13]. Protein hydrolysates produced from BSG not only exert antioxidant activity under conventional chemical assay but also exert antioxidant and immunomodulatory effects to cell lines (U937, Jurkat T cell, Caco-2 and HepG2) under oxidative stress *in vitro* [12, 14]. Consequently, a low cost, readily available BSG would make for an ideal material for the production of bioactive compounds. Furthermore, our previous study proved that the BSG residue resulting from protease-aided protein removal can be potentially used as a viable fibre source for ruminant feeding [15], thus increasing the by-product value.

Antibiotics, widely used in the north American beef industry, are of growing concern for consumers because of potential antibiotic residue accumulation in animal products and the increasing risk of antimicrobial resistance which will thus limit their future use as growth promotants in cattle feed. As a result, alternatives to in-feed antibiotics, using natural sourced materials, have been an area of significant research in recent years. Protein hydrolysates of various origins, including animal or agriculture production by-products, have been reported to possess antimicrobial activity [16–18], and have the potential to be used as alternatives to antibiotics. Protein hydrolysates of BSG have been reported to possess antibacterial activity towards *Escherichia coli* O157:H7 [19] and *Staphylococcus aureus* [20]. In an *in vitro* screening study, the hydrolysates derived from BSG protein decreased feed degradability with the response varying with type of proteases used in hydrolysates separation, hydrolysate dose rate and type of feed incubated [21]. The authors hypothesized that inclusion of BSG protein hydrolysates may exert antimicrobial activity in rumen environment and that protein hydrolysates of BSG could be used as functional feed additives to substitute antibiotics in ruminant diets. The objective of this study was to determine if BSG protein hydrolysates prepared with two different proteolytic enzymes (alcalase and flavourzyme) had the ability to improve nutrient disappearance and rumen fermentation, reduce methane production and alter the microbial community compared with monensin in rumen simulation technique (RUSITEC) fed a high-grain diet.

## Methods

### Preparation of BSG protein hydrolysates

The BSG protein hydrolysates used in the current study were prepared following alkali extraction, acid precipitation and enzyme hydrolysis as previously reported [11, 13]. In brief, BSG was milled through a 0.5-mm screen (Wiley Mill; standard model 4; Arthur Thomas Co., Philadelphia, PA, USA) solubilized in 0.1 mol/L NaOH at 20% (w/v) rate, followed by continuous stirring at 350 r/min at 50 °C for 2 h for protein extraction. The supernatant was then collected by centrifuging at 8000×*g* for 15 min at 20 °C, passed through an ultrafiltration membrane to obtain the protein fraction > 1 kDa which was further precipitated by acid (adjusting pH to 3.5), centrifuged at 8000×*g* for 15 min at 20 °C and then freeze dried. The obtained protein was dispersed in deionized water to reach a 5% (w/v) solution, hydrolyzed by 1% alcalase and flavourzyme at their optimum pH and temperatures (pH 8.0, 55 °C and pH 6.6, 50 °C, respectively). At the end of hydrolysis, all of the hydrolysate solutions were adjusted to pH 7.0, heated at 95 °C for 5 min to deactivate the enzyme and centrifuged at 8000×*g* for 30 min to separate the solubilized peptides and amino acids from the non-soluble substrates. The obtained hydrolysates with alcalase and flavourzyme were referred to AlcH and FlaH, respectively. The protein content and sample degree of protein hydrolysis were determined as described [11, 13] and results were shown in Table 1. The antioxidant properties, including 1,1-diphenyl-2-picryl hydrazyl (DPPH) free radical scavenging activity, ferrous ion chelating activity and reducing power were measured using chemical methods (Supplemental material S1).

**Table 1:** Solubility and degree of hydrolysis of brewers’ spent grain (BSG) protein hydrolysates generated using alcalase and flavourzyme

Item	BSG	AlcH	FlaH	SEM	*P* <
BSG protein extraction, % of DM	31.7	70.0	70.0	…	…
Solubility, %	37.5^b^	89.1^a^	86.5^a^	2.35	0.01
Degree of hydrolysis, %	…	34.7^a^	11.9^b^	5.37	0.01

*AlcH* Alcalase hydrolysates; *FlaH* Flavourzyme hydrolysates

^a, b^ Means within a row with different superscripts differ (*P* < 0.01)

### Experimental design and treatments

The experiment was a completely randomized design with four treatments assigned to sixteen fermentation vessels in two units of RUSITEC apparatus. Treatments were: 1) basal diet without hydrolysates or antibiotics (Control), 2) basal diet + 1% AlcH (AlcH), 3) basal diet + 1% FlaH (FlaH), and 4) basal diet + 33 mg monensin + 11 mg tylosin/kg diet DM (positive control; ANT). The basal diet contained 10% barley silage, 87% dry-rolled barley grain, and 3% vitamin and mineral supplement (DM basis; Table 2), The diet was prepared as total mixed ration, and grounded through a 4-mm sieve (Arthur Thomas Co., Philadelphia, PA, USA). Approximately 10 g (DM) of diet was weighed into nylon bags (10 cm × 20 cm; pore size of 50 μm, Ankom Technology Corp., Macedon, NY, USA). The protein hydrolysates and antibiotics were added to bags at the desired amount (DM basis) and manually mixed. The experiment was 15 d in duration, with 8 d for adaptation followed by 7 d for sampling and data collection.

**Table 2:** Ingredient and chemical composition of experimental diets

Item	Contents
Ingredient, %	
Barley silage^a^	10.0
Barley grain^b^, ground	87.0
Supplement^c^	3.0
Chemical composition, % of DM	
DM	91.6
OM	95.2
CP	11.8
NDF	20.0
ADF	8.0
Starch	50.3

^a^ Composition (DM basis): 31.8% DM, 94.1% OM, 42.9% NDF, 26.7% ADF, 16.4% starch, and 9.6% CP

^b^ Composition (DM basis): 90.2% DM, 98.4% OM, 14.9% NDF, 4.1% ADF, 55.9% starch and 12.4% CP

^c^ Supplied per kilogram of dietary DM: 565 g barley grain, 100 g canola meal, 250 g calcium carbonate, 25 g molasses, 30 g salt, 20 g urea, 0.66 g vitamin E 500 and 10 g premix. The premix in the supplement contained per kilogram of dietary DM: 15 mg of Cu, 65 of mg Zn, 28 mg of Mn, 0.7 mg of I, 0.2 mg of Co, 0.3 mg of Se, 6000 IU of vitamin A, 600 IU of vitamin D, and 47 IU of vitamin E

### Inoculum donor

Three ruminally fistulated Aberdeen Angus cross cows (average, 768 ± 95.1 kg BW) offered a high grain diet containing 8.2% barley silage, 89.2% dry rolled barley grain, and 2.6% vitamin and mineral supplement (DM basis) were used as rumen inoculum donor. Two hours after morning feeding, solid and liquid contents were collected from four locations within the rumen of each cow via rumen cannula. The contents were immediately filtered through four layers of cheesecloth, pooled (4 L per cow) and pH recorded before adding into the fermenters. The experimental protocols were reviewed and approved by the Lethbridge Research and Development Centre Animal Care Committee and the cows were handled in accordance with the guidelines of the Canadian Council on Animal Care [22].

### Experimental procedure

The RUSITEC procedure was carried out using two units of RUSITEC apparatus, equipped with eight 920-mL anaerobic fermenters of each unit, as described previously [23, 24]. In brief, to initiate the fermentation each fermenter was filled with 200 mL of McDougall’s buffer [25] and 700 mL of prepared rumen inoculum. Then, one bag containing 20 g of prepared solid rumen digesta and one bag containing 10 g of experimental diet (DM basis) were placed in each fermenter at 09:00 h on d 0. Fermenters were placed in a circulating water bath at 39 °C for the duration of the incubation period. After 24 h of incubation, the bag containing solid rumen digesta was replaced with a bag containing experimental diet. Thereafter, a feed bag was replaced daily, so that each bag remained in each fermenter for 48 h. During the daily feed-bag exchange, the fermenter was flushed with N_2_ gas to maintain anaerobic condition in the fermenters. The artificial saliva was continuously infused into fermenters using a peristaltic pump (Model ISM 932D, Ismatec, Index Health and science GmbH, Wertheim, Germany) at a dilution rate of 2.9%/h. Effluent and fermentation gasses from each fermenter were collected, respectively, into a 2-L Erlenmeyer flask and a reusable 2-L gas-tight collection bag (CurityR; Conviden Ltd., Mansfield, MA, USA) and the volume recorded at the feed bag exchange.

### Nutrient disappearance

Disappearance of DM, OM, CP, acid detergent fiber (ADF), neutral detergent fiber (NDF) and starch was measured with 48-h incubated feed bags from d 9 to 13 of the sampling period. Bags were withdrawn from fermenters and washed manually under running cold water until the water was clear and dried at 55 °C for 48 h (AOAC, 2005; method 930.15) to determine DM disappearance. Thereafter, the bag residues were pooled over 5 d by fermenter and ground through a 1-mm sieve for DM, OM, NDF and ADF analysis. A portion of ground sample was further ground using a ball mill (Mixer Mill MM2000; Retsch, Haan, Germany) for total N and starch analysis. Disappearance of DM, OM, CP, NDF, ADF and starch was calculated as the differences between the amount of input and the amount remaining of each nutrient in the residues.

### Gas production and dissolved gases

Fermentation gasses were measured every 24 h using a gas meter (Model DM3A, Alexander-Wright, London, England, UK) from d 9 to 15 of the sampling period. A sample (20 mL) was obtained from each bag once daily for determining gas profiles. On d 14 and 15, fermentation liquid was sampled in duplicate (35 mL each) from each fermenter to determine the concentration of dissolved H_2_ (dH_2_) and dissolved CH_4_ (dCH_4_). The sampling procedure was carried out using two syringes as reported [26]. In brief, a 50-mL syringe containing 35 mL of fermentation liquids was connected to a 20-mL syringe filled with 5 mL of N_2_. The N_2_ was injected from the small syringe into the large syringe via a T tube and valve and the apparatus was vigorously shaken by hand. The entire gas phase was then transferred from the large syringe into the small one to determine the gas volume. Finally, the small syringe was removed from the T tube and 6 mL of gas was sampled for both gas and dissolved gas analyses using a Varian 4900 Gas Chromatograph (Agilent Technologies Canada Inc., Mississauga, ON, Canada).

### Fermentation parameters

The pH of fermentation fluid of each fermenter was measured daily using a pH meter (Orion model 260A, Fisher Scientific, Toronto, ON, Canada) at the time of feed-bag exchange. From d 9 to 13 of sampling period, effluent (5 mL) was sampled and preserved with 1 mL of 25% metaphosphoric acid for VFA analysis, with another 5 mL of effluent preserved with 1 mL of H_2_SO_4_ (1% vol/vol) for NH_3_-N analysis. All samples were well mixed and frozen at − 20 °C until analyzed. The production (mmol/d) of total VFA, individual VFA and NH_3_-N were determined based on daily effluent volume.

### Microbial protein synthesis

Bacteria in the fermenters were labeled using ^15^N. From d 7 to 15, 0.3 g/L (NH_4_)_2_SO_4_ in McDougall’s buffer was replaced with 0.3 g/L ^15^N-enriched (NH_4_)_2_SO_4_ (Sigma Chemical Co., St. Louis, MO, USA; minimum ^15^N enrichment 10.01 atom%). From d 9 to 15, the effluent was preserved by adding 3 mL of a sodium azide solution (20%; wt/vol) to each effluent flask. On d 14 and 15, daily volume of effluent from each fermenter was recorded and 35 mL was sampled and centrifuged at 20,000×*g*, 4 °C for 30 min to isolate liquid-associated bacteria (LAB). The obtained pellets were washed with deionized water and centrifuged three times (20,000×*g*, 30 min, 4 °C), suspended in distilled water, freezing and lyophilisation for determination of N and ^15^N.

Feed particle-associated (FPA) and feed particle-bound (FPB) bacterial fractions were measured from 48-h feed residues. After 48-h of incubation, feed bags were squeezed to expel excess liquids, placed individually in a plastic bag with 20 mL of McDougall’s buffer and processed for 1 min using a Stomacher 400 Laboratory Blender (Seward Medical Ltd., London, UK). Then, the liquid from each feed bag was squeezed into a 50-mL centrifuge tube, the feed residues were washed twice with 10 mL of McDougall’s buffer in each wash, and the washed buffer pooled with the squeezed liquid to obtain the FPA bacterial fraction. The washed feed residues were considered as the FPB bacterial fraction. The obtained FPA samples were centrifuged at 500×*g*, 4 °C for 10 min to remove large feed particles, the supernatant centrifuged (20,000×*g*, 30 min, 4 °C) to isolate bacterial pellets and further processed as described previously. Washed feed residues (FPB fraction) were dried at 55 °C for 48 h, weighed for DM determination, and ball ground (MM400; Retsch Inc., Newtown, PA, USA) for N and ^15^N analysis. Total microbial protein synthesis was estimated as the sum of LAB, FPA and FPB.

### Microbial community

The microbial communities of FPA samples were assessed through high-throughput sequencing. Total DNA was extracted from FPA samples (30 mg) using a QIAamp Fast DNA stool mini kit (Qiagen, Hilden, Germany), according to the manufacturer’s instructions. The quality and quantity of extracted DNA was measured using a NanoDrop spectrophotometer (Thermo Fisher Scientific, Waltham, MA, USA), then the extracted DNA was stored at − 20 °C until sequencing. The V4 hypervariable region of the archaeal and bacterial 16S rRNA gene was amplified using the modified 515-F and 806-R primers, with the PCR conditions and sequencing steps carried out as previously described by [27]. Briefly, the 16S rRNA gene amplicons were generated using a two-step PCR, and then the amplicons were subjected to Illumina paired-end library preparation, cluster generation, and sequenced on an Illumina MiSeq instrument (Illumina, Inc., San Diego, CA, USA).

The obtained 16S rRNA gene sequencing raw data were processed using QIME2 [28] and the R-package DADA2 (Version 1.4) denoise method as described. In brief, after removing primer sequences and truncating both the forward and reverse reads at 225 bp, quality control was done for the reads using the QIME2, with chimeric sequences identified and removed. Then, the richness (number of OTUs) and diversity (Shannon index) were calculated and non-metric multidimensional scaling (NMDS) was performed based on Bray-Curtis similarity distances using R packages vegan (Version 2.4.4; [29]) and phyloseq (Version 1.20.0; [30]). Fold change of ruminal bacterial at genus level with a threshold of 5% was analyzed using R-package Deseq2 [31].

### Chemical analysis

The chemical analysis of the feeds and feed residues were conducted in duplicate, and repeated when the CV for the replicate analysis was more than 5%. Analytical DM was measured by oven drying at 135 °C for 2 h (AOAC, 2005; method 930.15) [32] and ash content was determined by combustion of samples at 550 °C for 5 h (AOAC, 2005; method 942.05), with OM content calculated as the difference between 100 and the ash content. The concentration of NDF (ash-free) was determined using a VELP Fiber Digestion System (VELP Scientifica, Burlington, ON, Canada) using the method of Van Soest et al. [33], with heat stable α-amylase (Termamyl 120 L, Novo Nordisk Biochem, Franklinton, NC, USA) and sodium sulfite included; while ADF was determined according to AOAC (2005; method 973.18). Total N of feed and residue samples and ^15^N of LAB, FPA and FPB samples were analyzed using combustion analyzer (NA 2100, Carlo Erba Instruments, Milan, Italy), with CP calculated as total N × 6.25. Starch was determined by enzymatic hydrolysis of α-linked glucose polymers as reported previously [34]. Concentration of VFA and NH_3_-N in the effluent was determined using a gas chromatograph (model 5890, Hewlett-Packard Lab, Palo Alto, CA, USA).

### Statistical analysis

Data were analyzed in a completely randomized design using the MIXED procedure of SAS (Version 16.0.0, SAS Inst. Inc., Cary, NC, USA), with treatment considered as a fixed effect, day of sampling as repeated measures, and the fermenter and RUSITEC apparatus as random effects. For the repeated measures, various covariance structures were tested with the final structure chosen based on the lowest Akaike’s information criteria value. Results are reported as least squares means, which were compared using the Tukey correction for multiple comparisons. Significance among treatments was declared at *P* ≤ 0.05 and a trend at 0.05 < *P* ≤ 0.10 unless otherwise stated.

## Results & discussion

### BSG protein hydrolysates of AlcH and FlaH

The protein content of BSG protein extract was 70%, which was much higher than that of the original BSG substrate (31.7%; Table 1). The present value was comparable to a recent study that utilized a combination of enzyme and ultrasonication extraction, resulting in 69.8% of BSG protein content [35]; whereas, it was greater than BSG pre-treated with carbohydrases followed by direct hydrolysis using proteolytic enzymes (63.1%) [36]. The degree of hydrolysis (DH) of the BSG protein was higher (*P* < 0.01) when treated with alcalase (34.7%) than with flavourzyme (11.9%), with both protein hydrolysates showing high solubility in water. The DH expresses the number of peptide bonds cleaved as a percentage of total number of peptide bonds in the substrate, with the higher DH expected to have lower molecular weight peptides and vice versa. Enzymatic hydrolysis is gaining importance in producing protein hydrolysates due to its high yield and products with more consistent quality under controllable hydrolysis conditions [17]. Alcalase and flavourzyme are commercially available enzymes that have been widely studied for generating bioactive peptides of various protein origin [11, 37]. Bamdad et al. [11] showed antioxidant activities of barley hordein and glutelin enzymatic hydrolysates generated with flavourzyme and alcalase. In the current study, BSG protein hydrolysates FlaH and AlcH had DPPH (1,1-diphenyl-2-picryl hydrazyl) free radical scavenging activity, Fe^2+^-chelating ability and superoxide radical scavenging capacity (Supplemental material S1). The peptides high in hydrophobic and non-polar amino acid residues were most likely responsible for the antioxidant effects [11, 13]. As the hydrophobic and non-polar amino acids mostly remain in BSG after the brewing process [11], it was hypothesized that the BSG hydrolysates may have beneficial effects in rumen feeds based on their antioxidant activities.

### Effects of AlcH, FlaH and ANT on nutrient disappearance and fermentation characteristics

Supplementation of FlaH reduced (*P* < 0.01) the disappearance of DM, OM, CP and starch compared to the control and AlcH, which in turn did not differ (Table 3). The reduction of DM disappearance by FlaH is consistent with the results obtained in our previous study using batch culture technique [21]. It that study we reported that the DM disappearance of barley grain linearly decreased from 78% to 67% as the inclusion rate of FlaH increased from 0, 0.5%, 1.0% to 1.5%. The reduction in the disappearances of DM and OM by FlaH primarily resulted from the decreased CP and starch, suggesting antimicrobial activity of protein hydrolysates. However, it appeared that the effect of AlcH and FlaH on the nutrient disappearances was not associated with their antioxidant activities and degree of hydrolysis measured in the present study. In fact, the increased DPPH scavenging or Fe-chelating activity appeared to adversely affect nutrient disappearance as observed with FlaH. It would be expected that the nutrient disappearance with AlcH would be less than that with FlaH because of the greater DPPH scavenging or Fe-chelating activity of AlcH than FlaH. These results suggest that the hydrolysate with chemically determined antioxidant activity may have different responses under rumen fermentation conditions. Furthermore, the fermentation in the RUSITEC system would have less oxidative stress compared with in vivo where oxygen can frequently enter the rumen with feeds and rumination. It also speculated that the hydrolysates may have more antimicrobial than antioxidant activity in the fermentation. Different bioactivities of AlcH and FlaH in rumen fermentation would be expected given that the activity of protein hydrolysates depends on the specificity of the protease used, hydrolysis conditions and degree of hydrolysis [13]. The flavourzyme is an endo- and exopeptidase enzyme mixture mainly producing small peptides and free amino acids, while the alcalase is an endo-protease mainly generating small- and medium-sized peptides [13]. Furthermore, about 6% of CP in the total protein input was hydrolysate origin as a result of adding FlaH, the decreased CP disappearance with FlaH could be due to the resistance of hydrolysate to microbial degradation. The resistance is related to the amino acid sequences and structures, especially peptides with Gly-Gly, Pro-X or X-Pro residues at the N-terminus, or with modification of N-terminal amino groups [38, 39]. Thus, it is hypothesized that FlaH generated by flavourzyme is more resistant to rumen microbial digestion and possesses strong bioactivity in modulating rumen fermentation including reducing the activity of proteolytic and starch utilization microbes. The disappearance of NDF and ADF were not affected by supplementation of either AlcH or FlaH, suggesting that both protein hydrolysates had limited effect on the activity of fibrolytic microbes.

**Table 3:** Effect of brewers’ spent grain (BSG) protein hydrolysates or antibiotics on nutrient disappearances and fermentation characteristics in RUSITEC

Item	Treatments^d^				SEM	*P* <
	Con	AlcH	FlaH	Ant		
Nutrient disappearance, %						
DM	76.5^a^	76.1^ab^	75.3^b^	72.7^c^	0.58	0.01
OM	77.7^a^	77.6^a^	76.5^b^	74.1^c^	0.56	0.01
CP	75.1^a^	75.4^a^	73.4^b^	69.7^c^	0.63	0.01
NDF	40.3^a^	38.7^a^	38.9^a^	31.8^b^	1.86	0.01
ADF	29.2^a^	26.8^a^	27.2^a^	21.5^b^	1.88	0.01
Starch	90.7^a^	90.8^a^	89.5^b^	87.8^c^	0.50	0.01
Fermentation characteristics						
pH	5.74^b^	5.78^b^	5.77^b^	5.85^a^	0.02	0.01
NH_3_-N, mmol/d	3.66^b^	4.05^a^	4.19^a^	2.57^c^	0.12	0.01
Total VFA, mmol/d	54.80^a^	54.51^a^	53.63^a^	48.88^b^	0.86	0.01
Individual VFA, % of total VFA						
Acetate (A)	30.97^a^	31.12^a^	31.67^a^	29.77^b^	0.38	0.03
Propionate (P)	39.33^b^	39.39^b^	39.42^b^	42.98^a^	0.39	0.01
Butyrate	20.37^a^	19.98^a^	19.20^b^	16.76^c^	0.45	0.01
BCVFA^e^	2.21^a^	2.19^a^	2.19^a^	1.56^b^	0.03	0.01
Valerate	4.25^b^	4.33^b^	4.78^b^	7.12^a^	0.41	0.01
Caproate	2.64^a^	2.77^a^	2.80^a^	1.57^b^	0.17	0.01
A:P	0.79^a^	0.79^a^	0.80^a^	0.69^b^	0.01	0.01

^a, b, c^ Least square means within a row with different superscripts differ (*P* < 0.05)

^d^ *Con* Control, no antioxidant peptide and no antibiotics, *AlcH* Alcalase hydrolysates, 10 mg per gram of TMR (DM basis), *FlaH* Flavourzyme hydrolysates, 10 mg per gram of TMR (DM basis), *Ant* antibiotics, 0.8 mg monensin + 1 mg tylosin per gram of TMR (DM basis)

^e^ Branched-chain volatile fatty acids (isobutyrate + isovalerate)

The pH in the fermenters remained constant throughout the whole experimental period, with higher (*P* < 0.01) pH with ANT than other treatments (average, 5.76; Table 3). Production of total VFA (mmol/d) and individual VFA molar proportion as well as acetate to propionate ratio were not affected with AlcH or FlaH supplementation except for a lower (*P* < 0.01) proportion of butyrate with FlaH compared to the control and AlcH. The similar VFA production is consistent with the biologically minor difference in OM disappearance despite of statistical difference between FlaH and control or AlcH. A higher proportion of propionate than acetate (+ 25%) was observed for all treatments, thus the ratio of acetate to propionate was below 1 (ranging from 0.69 to 0.80). The low ratio of acetate to propionate (< 1) would not be observed *in vivo* normal rumen condition, however it may *in vitro*. Russell [40] conducted an *in vitro* study to evaluate the effect of fermentation pH on acetate to propionate ratio and observed that the ratio for cracked corn incubations decreased from 1.2 to 0.6 when the final pH decreased from 6.5 to 5.3, thus indicating a reducing effect of low pH. Supplementation of AlcH and FlaH had higher (*P* < 0.01) NH_3_-N production than control. The higher NH_3_-N production with FlaH was unexpected due to the decreased CP disappearance by FlaH. This may reflective of their provision of a certain amount of peptides and free amino acid to fulfil partial nitrogen requirements of ruminal microbes. It has been reported that ruminal microbes prefer to utilize peptides or amino acids as a source of nitrogen or as a source of energy, thus leading to an accumulation of NH_3_-N [41]. It is speculated that the supplementation of AlcH and FlaH can promote the deaminative activity of high ammonia-producing bacteria. Ammonia is mainly produced by the low activity species but proliferation of the high ammonia producing bacteria can occur if certain diets were fed [38]. It has been reported that a variety of ruminal bacteria produce ammonia from protein hydrolysates, with strains of *Bacteroides ruminicola*, *Megasphaera elsdenii*, and *Selenomonas ruminantium* being the most active [42].

The supplementation of antibiotics (*P* < 0.01) decreased disappearance of DM, OM, CP, NDF, ADF and starch compared with the other treatments. Additionally, lower (*P* < 0.01) total VFA and higher (*P* < 0.01) fermenter pH was observed in the ANT treatment. Furthermore, ANT altered the individual VFA proportion compared to the other treatments, with lower (*P* < 0.01) molar proportion of acetate, butyrate, BCVFA and caproate, and higher (*P* < 0.01) molar proportion of propionate and valerate, thus led to lower (*P* < 0.01) acetate to propionate ratio. The supplementation of monensin and tylosin in the ANT treatment reduced (*P* < 0.01) the production of NH_3_-N compared to the other treatments, which was in alignment with previous reports that ionophores could decrease its production by suppressing the high NH_3_-N, producing microbial population, as well as the peptidelytic and deaminative activity of the bacteria that grow in the presence of ionophores [43]. Although FlaH had similar effects on reducing CP and starch disappearance to ANT, fibre disappearance and fermentation pattern were not affected. It is likely that mode of action in the rumen is different between monensin and FlaH. And the addition of FlaH protects protein and starch from rumen fermentation, facilitating more protein entering the small intestine for digestion and may alleviate risk of rumen acidosis.

### Effects of AlcH, FlaH and ANT on gas and dissolved gas production

During fermentation H_2_ is produced during the oxidation reduction of electrons by membrane-bound hydrogenases of H_2_ generating rumen microbes. This plays an important role in maintaining the oxidation-reduction homeostasis to guarantee rumen anaerobic fermentation [44]. However, the activity of membrane-bound hydrogenase will be inhibited under high H_2_ pressure conditions in the rumen. Whilst the methanogenic Archaea can efficiently use H_2_ to reduce CO_2_ to keep a low ruminal H_2_ pressure, they also producing enteric CH_4_ [45]. Therefore, either reducing the production of H_2_ or finding alternative sinks for H_2_ may reduce CH_4_ emissions. It is now clear that ionophore antibiotics, like monensin, can decrease CH_4_ production via inhibition of growth of H_2_ producing bacteria without causing side effects to succinate- and propionate-producing bacteria [43]. In the current study, the fermentation liquid and gases were analyzed to determine dissolved gas (dH_2_ and dCH_4_) and gas (H_2_ and CH_4_) production, respectively. Production of dissolved gas, total gas (L/d) and dH_2_ were not affected by treatment, whereas the production of dCH_4_ (% of dGas, mg/d or mg/g digested DM), and CH_4_ (% of gas) were lower (*P* < 0.01) with FlaH than with the control and AlcH which in turn did not differ (Table 4). Supplementation of either AlcH or FlaH decreased (*P* < 0.01) the production of H_2_ expressed as μg/d or μg/g digested DM. The ANT treatment had consistently lower (*P* < 0.05) production of CH_4_, H_2_ and dCH_4_ compared with the other treatments. The reduced production of H_2_ by adding FlaH (− 49%) is of interest and is consistent with the decrease in dCH_4_ production and the proportion of CH_4_ in the total gas. However, the molar proportion of propionate was unchanged by adding FlaH despite the decreased CH_4_ and H_2_. This may be explained by the decrease in OM disappearance with FlaH potentially unable to produce sufficient H_2_ to increase propionate production. These results suggest that the FlaH may be an effective means to mitigate enteric methane production arising from ruminal fermentation. The rationale for decreased production of H_2_ with AlcH without altering the production of CH_4_ and propionate however is unclear. The present results confirmed the effect of monensin on reducing CH_4_ and H_2_ production and increasing propionate production which has been observed in previous studies [43]. Although the FlaH treatment showed reduction of CH_4_ and H_2_ as well as the reduction in nutrient disappearance, the magnitude of the reduction was much less than the ANT treatment, and FlaH did not increase propionate production or alter fermentation patterns. Therefore, it suggests lower activity of FlaH than the monensin in the ANT treatment and different mode of action between the two additives. Future work also needs to be carried out to determine relationships between their physicochemical and techno-functional properties.

**Table 4:** Effects of brewers’ spent grain (BSG) protein hydrolysates or antibiotics on gas and dissolved gas production in RUSITEC

Item	Treatments^d^				SEM	*P* <
	Con	AlcH	FlaH	Ant		
Dissolved gas^e^						
Total dGas, L/d	0.95	0.96	0.96	0.94	0.02	0.17
dCH_4_, % of dGas	0.72^a^	0.68^a^	0.63^b^	0.53^c^	0.04	0.01
dCH_4_, mg/d	4.42^a^	4.21^a^	3.86^b^	3.17^c^	0.23	0.01
dCH_4_, mg/g DM digested	0.59^a^	0.58^a^	0.53^b^	0.45^c^	0.03	0.01
dH_2_, μg/d	0.48	0.63	0.58	0.54	0.20	0.38
dH_2_, μg/g DM digested	0.06	0.08	0.08	0.07	0.01	0.39
Gas Production^f^						
Total gas, L/d	1.68	1.62	1.59	1.58	0.20	0.87
CH_4_, % of gas	2.47^a^	2.55^a^	2.11^b^	1.32^c^	0.12	0.01
CH_4_, mg/d	27.49^a^	27.15^a^	24.37^a^	14.92^b^	5.38	0.01
CH_4_, mg/g DM digested	3.80^a^	3.77^a^	3.49^a^	2.19^b^	0.72	0.01
H_2_, μg/d	105.1^a^	65.3^b^	54.3^b^	18.0^c^	11.01	0.01
H_2_, μg/g DM digested	14.6^a^	9.0^b^	7.6^b^	2.6^c^	1.58	0.01

^a, b, c^ Least square means within a row with different superscripts differ (*P* < 0.05);

^d^ *Con* Control, no antioxidant peptide and no antibiotics, *AlcH* Alcalase hydrolysates, 10 mg per gram of TMR (DM basis), *FlaH* Flavourzyme hydrolysates, 10 mg per gram of TMR (DM basis), *Ant* antibiotics, 0.8 mg monensin + 1 mg tylosin per gram of TMR (DM basis)

^e^ Sampled from d 14 to 15

^f^ Sampled from d 9 to 13

### Effects of AlcH, FlaH and ANT on microbial protein synthesis and microbial community

**Fig. 1:**
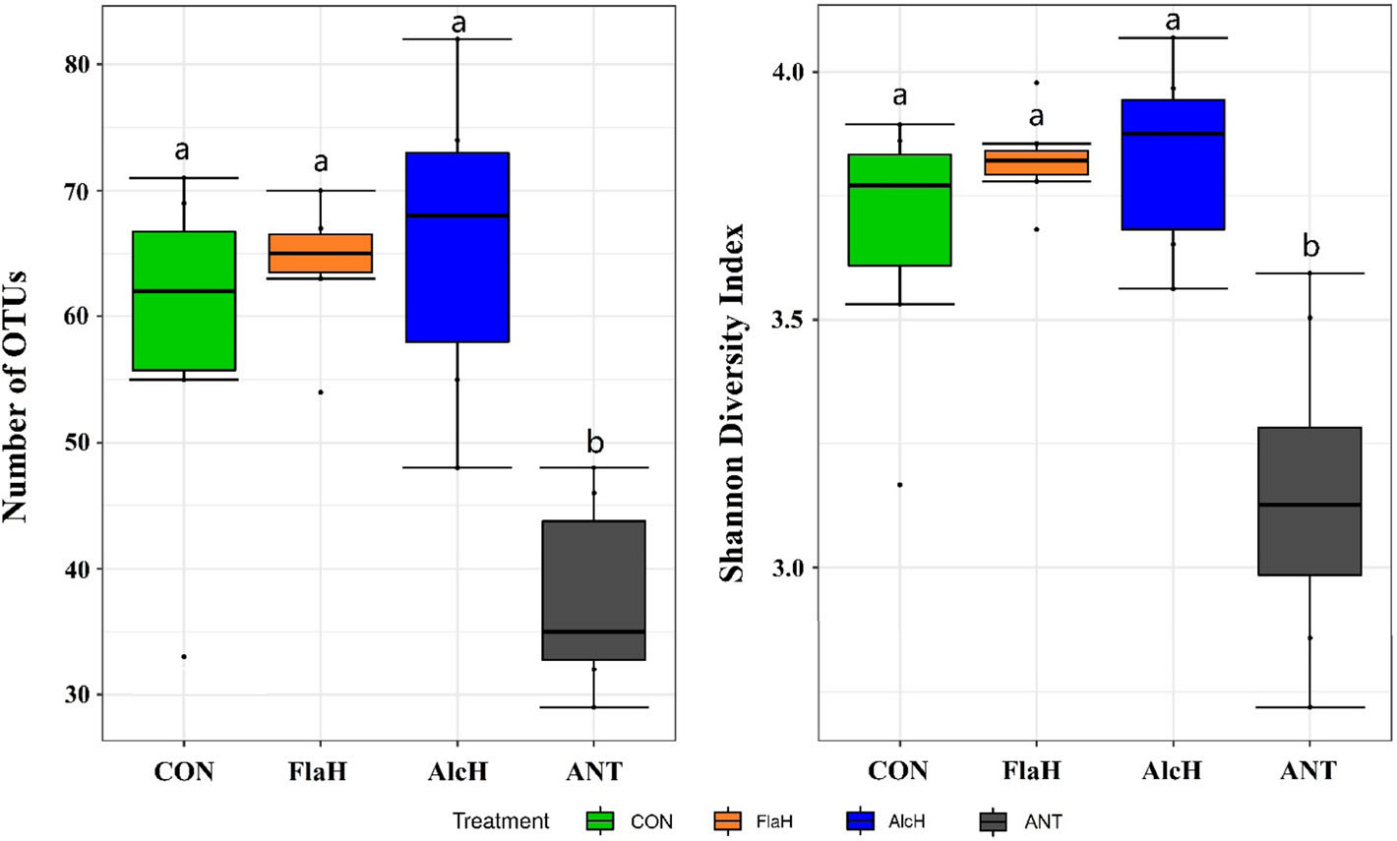
Box plots of the (**a**) number of OTUs and (**b**) Shannon diversity index for feed particle-associated (FPA) samples by treatment. Treatments were: control (CON), basal diet; FlaH, basal diet + 1% FlaH (dry matter basis; DM); AlcH, basal diet + 1% AlcH; ANT, basal diet + 33 mg monensin + 11 mg tylosin/kg diet DM. Different lowercase letters within each treatment indicate significantly different means (*P* < 0.05)

**Fig. 2:**
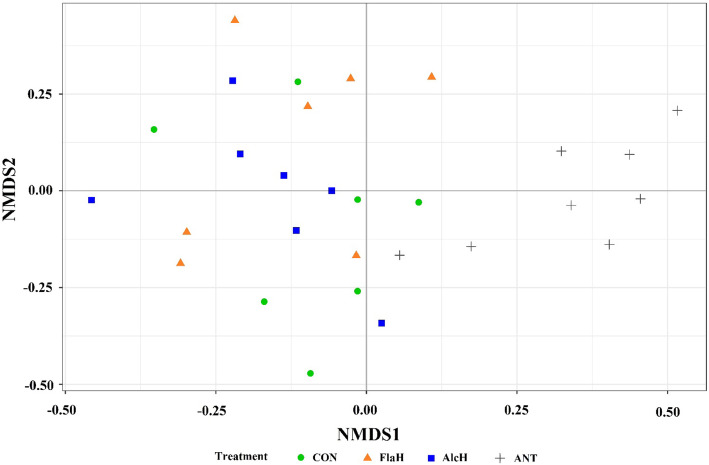
Non-metric multidimensional scaling (NMDS) plots of the Bray-Curtis dissimilarities for feed particle-associated (FPA) samples by treatment. Treatments were: control (CON), basal diet; FlaH, basal diet + 1% FlaH (dry matter basis; DM); AlcH, basal diet + 1% AlcH; ANT, basal diet + 33 mg monensin + 11 mg tylosin/kg diet DM. No statistical difference was obsevered among CON, FlaH and AlcH treatments, which were significantly (*P* < 0.05) different from ANT

Production of total microbial N (LAB + FPB + FPA) and LAB was less (*P* < 0.05) with FlaH than the control and AlcH treatments, whereas the production of FPA was higher (*P* < 0.01) with AlcH and FlaH with no difference in FPB among treatments (Table 5). The ANT treatment produced higher (*P* < 0.01) FPA but less LAB and total microbial N compared to the control and AlcH which were similar to FlaH except for LAB, which was less (*P* < 0.01) than ANT. Microbial N production efficiency did not differ among treatments. Although the total microbial N production was reduced with FlaH and ANT, the proportion of attached microbial biomass (FPA + FPB) in the total biomass was higher for these treatments (FlaH 30.1% and ANT 34.4%) than control (24.4%). Our results suggested that adding FlaH or ANT provides benefits for microbial colonization of feed particles. The supplementation of AlcH and FlaH to the basal diet increased the microbial population in the FPA fraction, which was interesting as microbial attachment is essential to process of feed digestion. Thus, the FPA samples were selected for high-throughput sequencing to assess the microbial community. Neither numbers of OTUs (Fig. 1a) nor Shannon diversity indices (Fig. 1b) of FPA microbial community were affected by supplementing AlcH and FlaH, whereas both indices were reduced (*P* < 0.05) by ANT. Similarly, the results of the NMDS analysis indicated that there was no specific clustering of the FPA microbial community in the control, FlaH or AlcH treatments, however the ANT treatment had a more independently clustered community structure (Fig. 2). Notwithstanding, the effects of BSG protein hydrolysates on ruminal microbiome are not fully understood. Meanwhile, our results revealed that monensin greatly reduced the diversity of FPA microbial community were in agreement with previous *in vitro* [46] and *in vivo* [47, 48] studies.

**Table 5:** Effect of brewers’ spent grain (BSG) protein hydrolysates or antibiotics on microbial N synthesis and microbial community of FPA in RUSITEC

Item	Treatments^d^				SEM	*P* <
	Con	AlcH	FlaH	Ant		
Production of microbial N^e^, mg/d						
LAB^f^	60.7^a^	60.7^a^	53.3^b^	48.2^c^	1.94	0.05
FPB^g^	7.0	6.2	6.4	7.0	1.04	0.51
FPA^h^	13.7^c^	16.6^b^	17.6^ab^	19.4^a^	1.82	0.01
Total	80.3^a^	82.5^a^	76.2^b^	73.5^b^	1.83	0.05
Efficiency of microbial protein^i^	7.0	7.1	6.9	6.9	0.26	0.45

^a, b, c^ Least square means within a row with different superscripts differ (*P* < 0.05)

^d^ *Con* Control, no antioxidant peptide and no antibiotics, *AlcH* Alcalase hydrolysates, 10 mg per gram of TMR (DM basis), *FlaH* Flavourzyme hydrolysates, 10 mg per gram of TMR (DM basis), *Ant* antibiotics, 0.8 mg monensin + 1 mg tylosin per gram of TMR (DM basis)

^e^ Samples from d 14 to 15

^f^ Liquid associate bacteria

^g^ Feed particle-bound bacteria

^h^ Feed particle-associated bacteria

^i^ Efficiency of microbial protein, mg microbial N production/g OM fermented

**Fig. 3:**
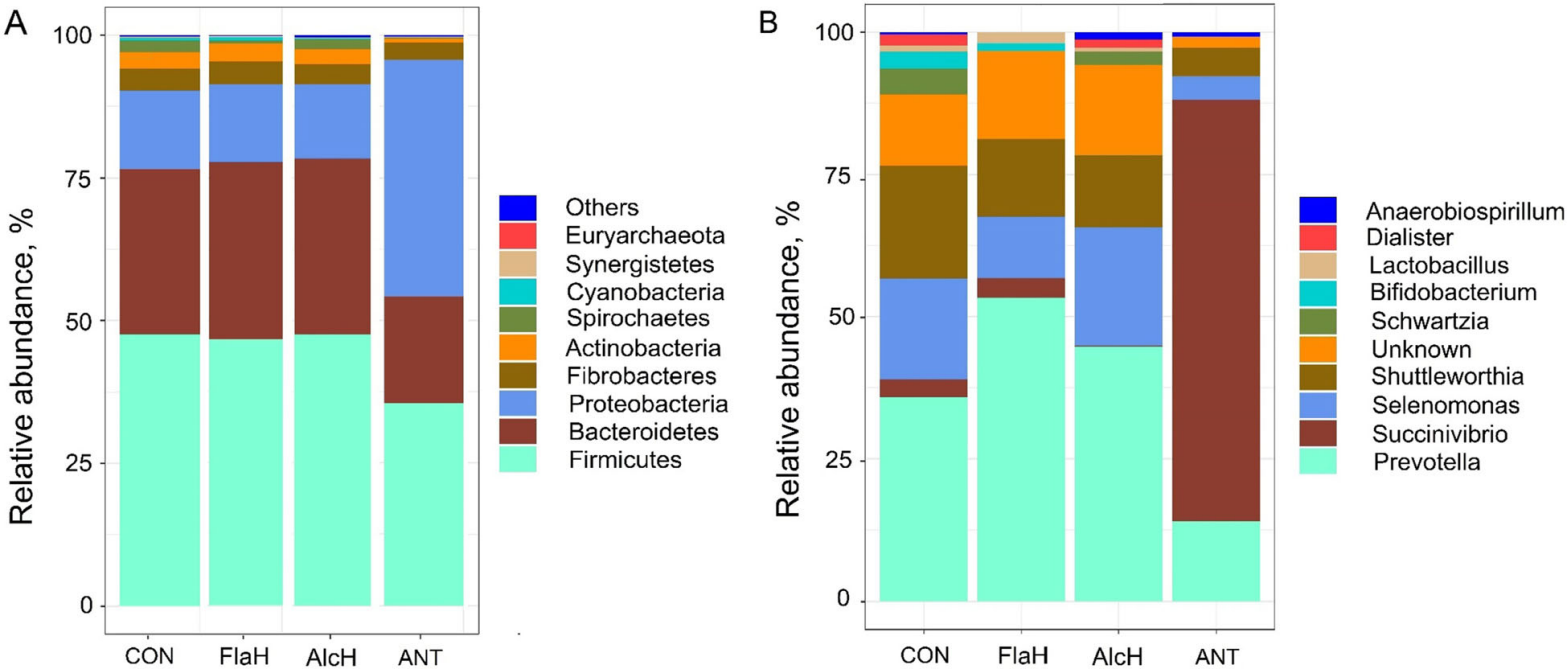
Effects of brewers’ spent grain (BSG) protein hydrolysates or antibiotics on the relative abundance (RA) of ruminal microbial at phylum (**a**) and genus (**b**) level. Treatments were: control (CON), basal diet; FlaH, basal diet + 1% FlaH (dry matter basis; DM); AlcH, basal diet + 1% AlcH; ANT, basal diet + 33 mg monensin + 11 mg tylosin/kg diet DM. The ANT reduced (*P* < 0.05) the RA of Firmicutes and Bacteroidetes and enhanced (*P* < 0.05) the RA of Proteobacteria at the phylum level; The FlaH treatment enhanced (*P* < 0.05) the RA of *Prevotella*, but reduced the RA of *Selenomonas*, *Shuttleworthia*, *Bifidobacterium* and *Dialister* as compared to the control. The AlcH treatment also had less (*P* < 0.05) RA of *Schwartzia* and *Bifidobacterium* than control. The ANT reduced (*P* < 0.05) the RA of *Prevotella*, *Selenomonas*, *Shuttleworthia*, *Bifidobacterium*, *Lactobacillus* and *Dialister*, and increased RA of *Succinivibrio* compared with control

**Fig. 4:**
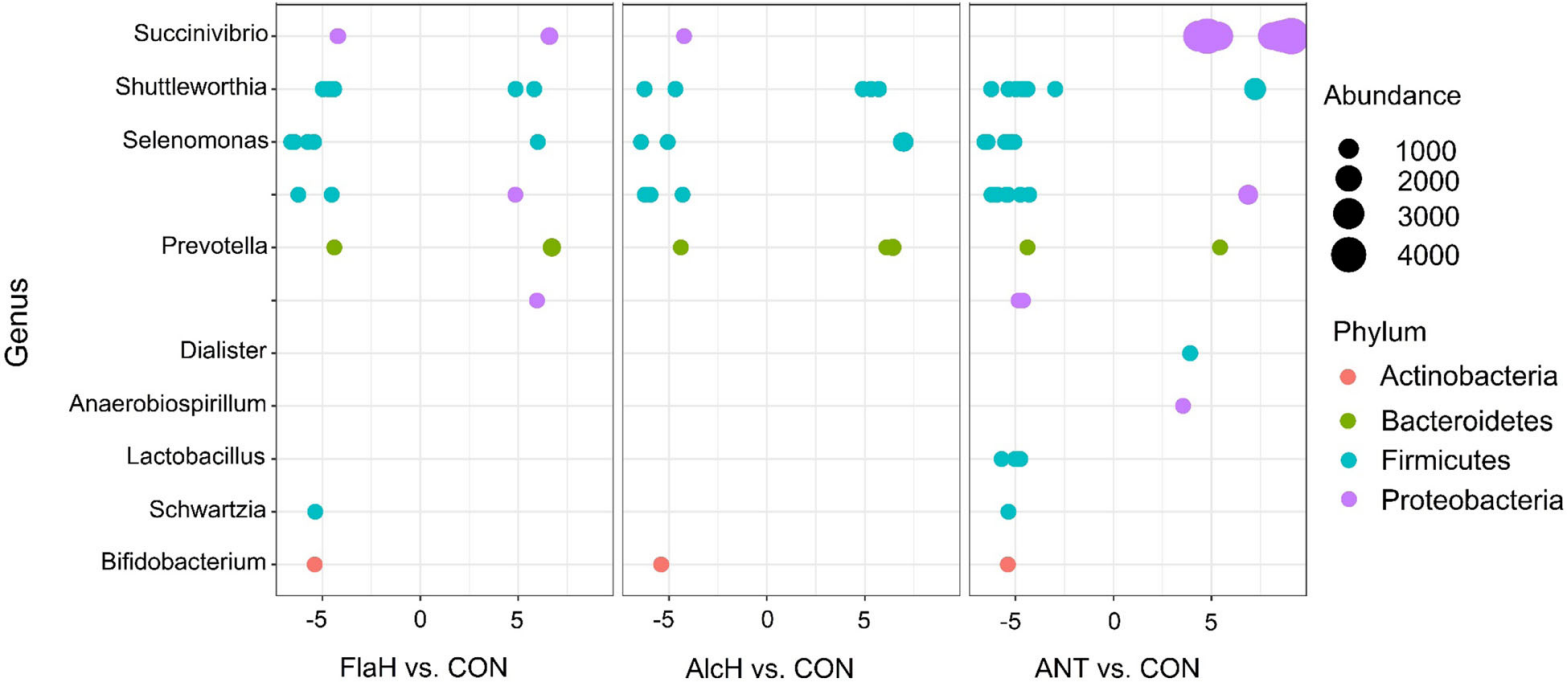
Relative changes (log_2_ fold; *P* < 0.05) of ruminal bacterial by treatment vs. control (CON) at genus level. Treatments were: CON, basal diet; FlaH, basal diet + 1% FlaH (dry matter basis; DM); AlcH, basal diet + 1% AlcH; ANT, basal diet + 33 mg monensin + 11 mg tylosin/kg diet DM

Analysis of the taxonomic composition revealed that AlcH and FlaH had no effect on the relative abundance (RA) of bacteria at the phylum level (Fig. 3a). However, the addition of antibiotics reduced (*P* < 0.05) the RA of Firmicutes and Bacteroidetes and enhanced (*P* < 0.05) the RA of Proteobacteria at the phylum level. For all treatments, phyla of Firmicutes, Bacteroidetes and Proteobacteria were the most predominant, accounting for approximately 90% of the microbial biomass in FPA, which was consistent with the sequencing results of liquid associated microbial community of *in vitro* [46] and *in vivo* studies [47, 48]. This suggested that phyla of Firmicutes, Bacteroidetes and Proteobacteria were predominant in both the liquid and particle-associated ruminal microbiome. While at the genus level, *Prevotella*, *Succinivibrio*, *Selenomonas*, *Shuttleworthia*, *Schwartzia*, *Bifidobacterium*, *Lactobacillus*, *Dialister* and *Anaerobiospirillum* were among the 10 most abundant genera in FPA microbial community (Fig. 3b) and should be considered as the “core bacteria” associated with feed particles. The FlaH treatment enhanced (*P* < 0.05) the RA of *Prevotella*, but reduced the RA of *Selenomonas*, *Shuttleworthia*, *Bifidobacterium* and *Dialister* as compared to the control. The AlcH treatment also had less (*P* < 0.05) RA of *Schwartzia* and *Bifidobacterium* than control. Notably, ANT reduced (*P* < 0.05) the RA of *Prevotella*, *Selenomonas*, *Shuttleworthia*, *Bifidobacterium*, *Lactobacillus* and *Dialister*, and increased RA of *Succinivibrio* compared with control. Furthermore, the Log_2_ fold change analysis found that several genera from phyla Actinobacteria, Bacteroidetes, Firmicutes and Proteobacteria had 5% more change (enhanced or reduced; *P* < 0.05) than the control (Fig. 4). Additionally, genera of *Shuttleworthia*, *Selenomonas*, *Lactobacillus* and *Schwartzia* from phyla Firmicutes were especially susceptible to FlaH, AlcH and ANT supplementation. These results indicated that FlaH and AlcH had less antibacterial effects compared to ANT. As *F. succinogenes*, *R. albus*, *R. flavefaciens*, *P. ruminicola*, *E. cellulosolvens*, and *E. ruminantium* are major rumen fibrolytic bacteria [49], the present results indicated that the application of BSG protein hydrolysates had no obvious detrimental effect on fibrolytic bacteria. This was supported by no treatment effect of FlaH and AlcH on the disappearances of NDF and ADF.

More susceptible to FlaH and AlcH were genera of *Shuttleworthia*, *Selenomonas*, *Lactobacillus* and *Schwartzia* from phyla Firmicutes and genera of *Bifidobacterium* from phyla Actinobacteria thus suggesting that the BSG protein hydrolysates had antibacterial activity which were more potent to Gram-positive bacteria than to Gram-negative bacteria. Similar results were reported that indicated that Gram-positive bacterial strains used were more sensitive to whey hydrolyzed by Alcalase than Gram-negative bacterial strains [50]. The specific mechanism is unknown, however it appears that this difference might be attributed to the lack of protective outer membrane in Gram-positive bacteria [51]. The exhibited antibacterial activity of FlaH and AlcH against the above-mentioned rumen bacteria is probably due to the release of certain antibacterial peptides upon Flavorzyme and Alcalase hydrolysis of the BSG proteins. The antibacterial peptides exert antibacterial effects due to their structure of alternating hydrophobic and cationic portions [52], with the cationic component likely able to bind to the polar heads of the negatively charged membrane phospholipids of bacteria. Meanwhile, the hydrophobic components are then inserted into the cell membrane of the bacteria, thus destabilizing water inlet and causing cell lysis. This may be further explained if looking at essential oils with antioxidant activity as they had stronger antibacterial activity than other essential oils [53], thus it may be speculated that the antibacterial effects of BSG protein hydrolysates might also be because of their antioxidant activities. The antioxidant activity of protein hydrolysates relies on the enzymes and methods used during BSG protein hydrolysis. Similarly, differences in effectiveness of monensin on ruminal Gram-positive and Gram-negative bacteria were also observed in the current study, and confirm the monensin sensitivity to Gram-positive bacteria [47].

The rumen microbial community is usually dominated by *Prevotella* at genus level when fed high grain diets [54]. Members of *Prevotella* are able to utilise various nutrients such as starch, proteins and non-cellulosic polysaccharides [55, 56], and to convert lactate into propionate to prevent accumulation of the former [48]. Moreover, as *Prevotella* are also H_2_-consuming bacteria in addition to *Selenomonas* [57], the reduced H_2_ with FlaH and AlcH was likely due to the increased RA of genus *Prevotella*. Meanwhile, members of *Prevotella* are known as the predominant proteolytic bacteria with a great diversity of extracellular proteolytic activities in the rumen [38, 58], thus, the decreased disappearance of CP by supplementing monensin could be explained by decreased RA of *Prevotella*. Currently, probiotics such as direct-fed microbials are often developed from the genera *Lactobacillus* and *Bifidobacterium* [59] and the reduced relative abundance of *Lactobacillus* and *Bifidobacterium* with ANT suggested a disadvantage of applying monensin in the diet. The genus *Schwartzia* are asaccharolytic and can ferment succinic acid to produce propionic acid [60]. It was reported that bacteria affiliated with *Schwartzia* were negatively correlated with CH_4_ emissions [61], which was contrary to our results that monensin reduced the RA of Schwartzia and CH_4_ emissions.

There is limited information on the genus *Shuttleworthia*, and its function in the rumen is almost unknown [54]. Recent studies found that the genus *Shuttleworthia* are digesta-adherent rumen bacteria in dairy and beef cattle [62] and are starch and sugar utilizers [63]. It has been reported that supplementation of phytogenic compounds decreased the RA of *Shuttleworthia* in dry dairy cows [63]. In the current study, the decreased *Shuttleworthia* observed with the BSG hydrolysates and ANT treatments may explain the decreased starch digestibility with FlaH and ANT. A recent study has shown a negative correlation between *Shuttleworthia* and lactate and NH_3_-N concentration [64], thus explaining the higher NH_3_-N production with BSG hydrolysates.

Bacteria from genus *Selenomonas*, in addition to degrading starch and cellulose, play a critical role in maintaining normal rumen fermentation through conversion of lactate and succinate into propionate, reducing lactate accumulation [65–67] and consumption of H_2_ to maintain low rumen H_2_ concentration [68, 69]. As fumarate and nitrate reducers, *Selenomonas* were proven a significant H_2_ sink in sheep [70]. Ruminants fed high starch diets often display high rumen dH_2_ concentrations, which would promote the growth of *S. ruminantium* for removing of electrons derived from fermentation [68]. Therefore, in the present study, the reduced H_2_ production arising from supplementation of BSG protein hydrolysates or monensin is consistent with the reduced relative abundance of *Selenomonas*.

Members of genus *Succinivibrio* can ferment both starch and cellulose into succinate and succinate then fermented by *Selenomonas* and other bacteria into propionate [67]. Therefore, the abundance of members of *Succinivibrio* in the rumen has been reported to be positively associated with feed efficiency of ruminant livestock [71, 72]. The increased RA of *Succinivibrio* with monensin in the present study was correlated with the increased propionate proportion and the increased fermenter fluid pH. Our results agreed with a previous study that indicates that monensin can lead to an increase in abundance of succinate producers [47] and that *Succinivibrio* is positively correlated to ruminal pH [66, 73]. The increased pH with ANT would be partly due to the increased RA of genus *Dialister*, which was reported to play a role in altering the buffering capacity of rumen fluid [74]. Furthermore, although the effects of monensin on methanogens were not determined in this study, the inclusion of monensin reduced H_2_ production and CH_4_ emissions. Our results supported the theory that decreased methane production with ANT most likely resulted from the decrease in nutrient availability for methanogenesis by acting on other rumen microorganisms instead of a direct inhibition of the methanogens [47].

## Conclusion

Inclusion of FlaH with antioxidant activity to high grain diet showed potential to reduce ruminal CP and starch disappearance, without affecting fibre disappearance, fermentation pH, and VFA profiles. Supplementation of FlaH also decreased H_2_ production and suggested potential mitigation effects on CH_4_ production. The different effectiveness of FlaH and AlcH on altering *in vitro* rumen fermentation and gas production is likely due to the different pepetide compositions generated with different hydralysate enzymes. Our results suggested that FlaH had similar but lesser effects to monensin. Enzymatically hydrolysis protein extracts from BSG with flavourzyme is a feasible way of processing brewing by-products which can benefit both the brewing industry and animal production, as well reducing wastes and enteric methane emissions. Further research is needed to purify and identify bioactive peptides with antioxidant and antibacterial activities from BSG protein hydrolysates in addition to a metabolism or growth study will be carried subsequently at this research instituition.

## Supplementary Information

**Additional file 1.**

## Data Availability

The data analyzed during the current study are available from the corresponding author on reasonable request.

## Abbreviations

A:P: Ratio of acetate to propionate
ADF: Acid detergent fibre
AlcH: Protein hydrolysates generated under Alcalase hydrolysis
BCVFA: Branched-chain volatile fatty acids (isobutyrate + isovalerate)
BSG: Brewers’ spent grain
CP: Crude protein
DM: Dry matter
FlaH: Protein hydrolysates generated under Flavourzyme hydrolysis
FPA: Feed particle associated bacteria
FPB: Feed particle-bound bacteria
LAP: Liquid associated bacteria
NDF: Neutral detergent fibre
RA: Relative abundance
TMR: Total mixed ration
VFA: Volatile fatty acid
OM: Organic matter
