# Supplementary material for: Effects of brewers’ spent grain protein hydrolysates on gas production, ruminal fermentation characteristics, microbial protein synthesis and microbial community in an artificial rumen fed a high grain diet
Source: J Anim Sci Biotechnol. 2021 Jan 4;12:1. doi: 10.1186/s40104-020-00531-5 (PMC7780661; doi:10.1186/s40104-020-00531-5)
Supplement: Supplementary file 1 — Additional file 1. [file 40104_2020_531_MOESM1_ESM.docx]

**Supplementary Material**

**Title: Effects of brewers’ spent grain protein hydrolysates on gas production, ruminal fermentation characteristics, microbial protein synthesis and microbial community in an artificial rumen fed a high grain diet**

Reference:

Galhenage Thanusha Ranithri Abeynayake (2019) Development of hydrolysates with antioxidant effects from brewers’ spent grain proteins, MSc. Thesis supervised by Dr. Lingyun Chen, Department of Agricultural, Food and Nutritional Science, University of Alberta

**1. Preparation of BSG protein hydrolysates**

An aqueous suspension of 2% (w/v) BSG protein was hydrolyzed with alcalase or flavourzyme at 50^o^C for 4 h. The pH was 8 for alcalase treatment, and 6 for flavourzyme treatment. The substrate was mixed with 4% (w/w) enzyme (based on protein dry weight). The pH and temperature were monitored throughout the hydrolysis. At the end of hydrolysis, enzymes were inactivated by heating at 80^o^ C for 20 min. Centrifugation at 8,000×*g* for 15 min at 20^o^ C was done to separate solubilized protein from insoluble substances. The collected supernatant was adjusted to pH 7.0 and freeze dried.

**2. DPPH free radical scavenging activity**

For DPPH assay, one milliliter of samples (1.0 mg/mL) were mixed with 1 mL of 0.1 mmol/L DPPH in anhydrous ethanol. The mixture was shaken vigorously and incubated at room temperature for 30 min under light protection. BHT and ascorbic acid at concentrations of 0.1 and 0.01 mg/mL were used as positive controls. The reduction of DPPH free radicals was determined by measuring the absorbance at 517 nm. The ability of the samples to scavenge DPPH free radicals was calculated according to the following equation:

%inhibition of DPPH radicals = (A_0_-A)/A_0_ ×100 %

Where A_0_ and A represent the absorbance of the control and hydrolysate sample (control contained everything except hydrolysate), respectively.

**3. Superoxide radical scavenging activity**

Superoxide radical scavenging activity was measured by monitoring the inhibition of pyrogallol autoxidation and polymerization. Briefly, 80 µL of 2.0 mg/mL sample was mixed with 80 µL of 0.05 mol/L Tris-HCl buffer (pH 8.3) containing 1 mmol/L EDTA, followed by addition of 40 µL of 1.5 mmol/L pyrogallol in 1.0 mmol/L HCl. Absorbance was measured at 320 nm for 5 min at room temperature against the blank. Tris-HCl buffer was used instead of samples in the blank. Ascorbic acid at 0.01 and 0.1 mg/mL was used as the positive control. Superoxide radical scavenging activity was calculated using following equation:

Superoxide radical scavenging activity = [(∆A_0_/min) －(∆A/min)]/ (∆A_0_/min) × 100

Where A_0_ and A represent absorbance of the blank and the sample, respectively.

**4. Ferrous ion chelating activity**

For ferrous ion chelating activity measurement, 1 mL of 20 µmol/L FeCl_2_ was mixed with 0.5 mL of 1.0 mg/mL sample, and then 1 mL of 0.5 mmol/L ferrozine was added to initiate the reaction. Mixture was incubated at room temperature for 15 min prior to measuring absorbance at 562 nm. The EDTA at concentrations of 0.1 and 0.01 mg/mL were used as positive controls. Ferrous ion chelating ability was calculated by the following equation:

% Ferrous ion chelating ability = (B_0_－B)/B_0_ ×100 %

Where B_0_ and B represent the absorbance of the sample and the control (distilled water instead of hydrolysates), respectively.

**Table 1.** Antioxidant activity of brewers’ spent grain (BSG) protein hydrolysates generated using alcalase and flavourzyme

| Item | BSG | AlcH1 | FlaH^2^ | SEM | *P* value |
| --- | --- | --- | --- | --- | --- |
| DPPH radical scavenging activity, % | 66.8^c^ | 72.7^a^ | 70.6^b^ | 0.56 | 0.02 |
| Ferrous ion chelating activity, % | 16.9^c^ | 22.6^a^ | 19.8^b^ | 0.37 | 0.01 |
| Reducing power | 0.302^b^ | 0.405^a^ | 0.428^a^ | 0.018 | 0.01 |

^1^AlcH = Alcalase hydrolysates;

^2^FlaH = Flavourzyme hydrolysates

^a, b^ Means within a row with different superscripts differ (*P* < 0.05)
